# Familial Mediterranean Fever Complicated by a Triad of Adrenal Crisis: Amyloid Goiter and Cardiac Amyloidosis

**DOI:** 10.1155/2020/7865291

**Published:** 2020-05-19

**Authors:** Sadi A. Abukhalaf, Beesan W. Dandis, Tasnim Za'tari, Adham M. Amro, Tareq Z. Alzughayyar, Yazan A. Rajabi

**Affiliations:** ^1^Al-Quds University Faculty of Medicine, Jerusalem, Palestine, Israel; ^2^Medicine Department, Ahli Hospital, Hebron, Palestine, Israel

## Abstract

**Background:**

FMF is a common disease in the Mediterranean populations and may be complicated by AA amyloidosis. The coexistence of adrenal and thyroid amyloidosis in AA amyloidosis secondary to familial Mediterranean fever (FMF) is an extremely rare reported condition. We presented a previously unreported triad of adrenal, thyroid, and cardiac amyloidosis secondary to FMF. *Presentation of Case*. We reported a 23-year-old Palestinian male patient presented with hypotension, vomiting, diarrhea, and abdominal pain. The patient was subsequently diagnosed to have an adrenal crisis with both amyloid goiter and cardiac amyloidosis.

**Conclusion:**

It is crucial to recognize the adrenal crisis in patients with AA amyloidosis secondary to FMF who present similarly to acute FMF inflammatory episodes. The adrenal crisis has high morbidity and mortality, especially if not recognized early in the course of the disease.

## 1. Introduction

Familial Mediterranean fever (FMF) is an autoinflammatory disorder with autosomal recessive (AR) inheritance characterized by spontaneously resolving self-limited recurrent paroxysms of fever and serosal inflammation mainly presented as abdominal and chest pain [1]. The most devastating complication of FMF is AA amyloidosis in which amyloid proteins are deposited in several organs most commonly kidneys leading to end-stage renal disease (ESRD). Amyloid proteins are rarely deposited in the adrenal glands causing adrenal dysfunction and acute adrenal crisis, and in the thyroid gland causing amyloid goiter [2]. Although the coexistence of both amyloid adrenal crisis and amyloid goiter has rarely been reported [3], the triad of adrenal crisis, thyroid, and cardiac amyloidosis has not been reported. Herein, we report a rare case of a 23-year-old man with a 20-year history of FMF complicated by amyloidosis presented to the emergency department with adrenal crisis and clinically detectable enlarged thyroid gland with cardiac amyloidosis diagnosed by echocardiography. To date, this is the first reported case presented with the triad of adrenal crisis, thyroid, and cardiac amyloidosis.

## 2. Case Presentation

A 23-year-old Palestinian male patient presented to our emergency department due to one-day duration of vomiting, diarrhea, and fever. The patient had FMF since the age of 3 years. The FMF was diagnosed by clinical manifestations and supported by genetic testing. The genetic test showed that the patient was homozygous for the pathogenic M694V MEFV gene mutation. Family history was significant for genetically confirmed FMF in father, two brothers, and one sister. The genetic tests for the sick family members showed that all patients were homozygous for the pathogenic M694V MEFV gene mutation.

The patient was maintained on 0.5–1 mg per day colchicine though the patient was not compliant with the medication. Although the patient was maintained on daily colchicine, the patient had a high-variable frequency of attacks. The patient developed FMF attacks on an average of 6–11 months though with an increased frequency when the patient aged 12 years. The FMF attacks were used to present as fever and abdominal and joints pain. These attacks were managed by NSAIDs administration with no change in the dose of the daily colchicine. The patient had no clue about any consistent triggering events. The patient had a past surgical history of appendectomy at the age of 9 years.

Our patient had significant risk factors to develop AA amyloidosis included male gender, a positive family history, suboptimal daily dose of colchicine, and the patient's medication noncompliance. At the age of 17 years, the patient developed ESRD secondary to tissue-diagnosis amyloid nephropathy and started on regular hemodialysis and 2 mg per day colchicine. At the age of 21 years, the patient underwent right kidney transplantation and was started on immunosuppressants and continued the 2 mg per day regimen of colchicine. His current medications were 2 mg per day colchicine with good compliance, tacrolimus, prednisolone, and mycophenolate sodium. The sick family members were maintained on 1-2 mg per day colchicine with very good compliance and no signs of amyloidosis or renal disease.

**Figure 1:**
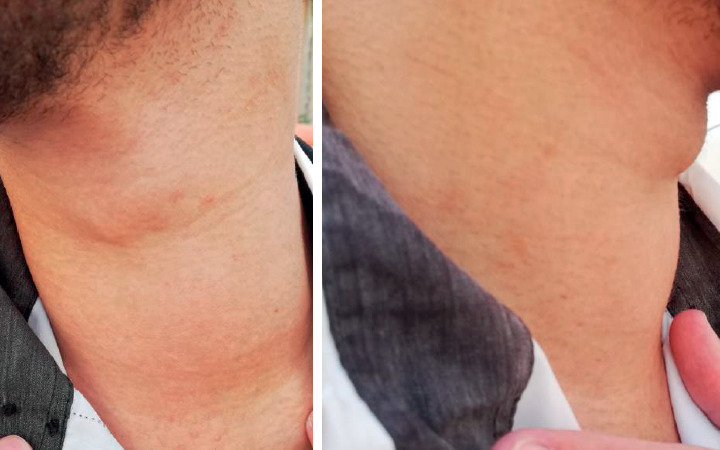
Physical neck examination photographs show enlargement of the thyroid gland.

Before admission, the patient had one-day duration of nausea, vomiting, diarrhea, weakness, and fever. He also complained of cough and chest tightness but no dysphagia. Physical examination showed an ill-looking, distressed and confused young patient with dry and pale mucous membranes and skin. The patient had no focal neurological deficits or meningeal signs. The neck was diffusely enlarged (Figure 1). There was no pigmentation of mucous membranes or skin. There was no history of fine tremor, heat or cold intolerance, increased or decreased appetite, and/or weight loss or gain.

Blood pressure, heart rate, and temperature were 50/30 mmHg, 110 beats/minute, 38.6°C, respectively. There were no added heart sounds or murmurs, or hepatosplenomegaly. Initial labs showed serum white blood cells count (WBC) of 34 × 10^9^/L, creatinine (Cr) of 1.6 mg/dL, blood urea nitrogen (BUN) of 23 mg/dl, sodium of 132 mEq/L, potassium of 5.3 mEq/L, magnesium of 1.3 mg/dl, and C-reactive protein (CRP) of 102 mg/L. An arterial blood gas (ABG) test showed pH of 7.2, partial pressure of carbon dioxide (PCO2) of 49 mmHg, partial pressure of oxygen (PO_2_) of 95 mmHg, and bicarbonate (HCO3) of 19 mmol/L. Thyroid function tests were normal. A low-cortisol serum level was noted (0.2 *μ*g/dl), but the adrenocorticotropic hormone (ACTH) stimulation test was inconclusive. Blood, stool, and urine cultures showed no pathogenic growths.

The patient was admitted to the intensive care unit (ICU) and received intravenous hydrocortisone, mineralocorticoid replacement with fludrocortisone, and aggressive fluid resuscitation. Antibiotics were also administered but discontinued given the negative cultures' results. Three days later, the patient was transferred to the medical ward where he was started on low-dose hydrocortisone and fludrocortisone.

**Figure 2:**
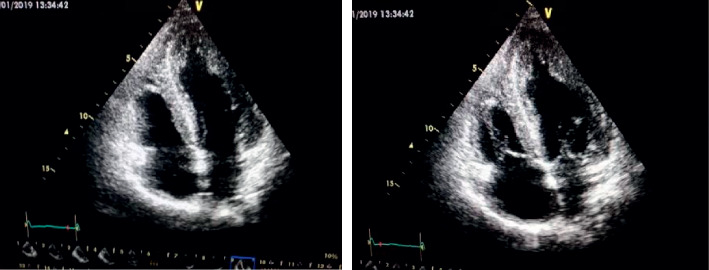
Echocardiogram photographs show granular sparkling of the myocardium walls suggesting cardiac amyloidosis.

Abdominal ultrasonography (US) showed a mild increase in cortical echogenicity of the transplanted kidney and bilateral atrophied kidneys with loss of cortico-medullary differentiation but otherwise was unrevealing. Echocardiogram showed normal size left ventricle (LV) and right ventricle, ejection fraction of 62%, moderate LV hypertrophy, mild diastolic dysfunction, normal size inferior vena cava (IVC), and no pericardial effusion. It also showed granular sparkling of the myocardium wall suggestive of cardiac amyloidosis (Figure 2). Neck US showed diffuse enlargement of both thyroid lobes and isthmus, retrosternal thyroid extension, and no enlarged lymph nodes. Fine needle aspiration (FNA) was negative for thyroid cancer and very suggestive for amyloidosis.

The patient's condition recovered rapidly and at the admission day 6, the patient did very well and was discharged home. During one year of follow-up, the patient did not develop any similar or significant conditions.

## 3. Discussion

Familial Mediterranean fever (FMF) is an AR disorder caused by Mediterranean fever (MEFV) gene mutations. The most frequent MEFV gene mutations in the Arabs ethnic group are V726A, M680l, M694V, M694l, and E148Q [4]. FMF can present differently in the same family members indicating the crucial roles of different genes involved, environmental factors, and gender.

FMF was first described in 1945 as “benign paroxysmal peritonitis” and also named as “recurrent polyserositis.” The diagnosis of FMF relies mainly on clinical findings based on different clinical diagnostic criteria supported by genetic testing [5, 6]. Genetic testing by confirming existence of two MEFV mutations is usually used to support the diagnosis of FMF, to exclude other disorders that may mimic FMF, and to counsel family members [7–9]. However, genetic testing for MEFV mutations is negative in 10%–20% of clinically diagnosed FMF patients and is positive for only one identifiable MEFV mutation (i.e, not diagnostic) in 33% of clinically diagnosed FMF patients [10, 11]. In case no MEFV mutations identified in patients who meet clinical diagnostic criteria, favorable response (i.e, relief of attacks and recurrence after colchicine cessation) to 6 months trial of colchicine treatment favors the diagnosis of FMF [8, 11].

Several diagnostic criteria were proposed, though the most frequently criteria used was developed at the Tel Hashomer Medical Center in Israel [12]. FMF may present with recurrent fever, abdominal pain, chest pain, joint pain, skin lesions and rash, exertional myalgia, acute pericarditis, acute scrotum, protracted febrile myalgia, headache, and aseptic meningitis. FMF diagnosis is supported in patients with family history of FMF, appropriate ethnic origin, age <20 years at disease onset, severe attack requiring bed rest, spontaneous remission of attack, symptom-free interval between attacks, attacks associated with transient inflammatory response with one or more abnormal laboratory results for white blood cell count, erythrocyte sedimentation rate, serum amyloid A, and/or fibrinogen, episodic proteinuria/hematuria, negative laparotomy or removal of normal appendix, consanguinity of parents, and favorable response to colchicine [12].

European league against rheumatism (EULAR) recommendations are the most up to date used guidelines in FMF management [13]. EULAR reported 18 recommendations and set the complete control of unprovoked attacks and minimizing subclinical inflammation in between attacks as the ultimate goal of treatment in FMF patients. In FMF attacks, the patient should be continued on the usual dose of colchicine and use NSAIDs. EULAR recommends treatment with colchicine as soon as an FMF diagnosis is made clinically with single or divided doses, depending on patient's tolerance and compliance. Physicians should increase the colchicine dose in the setting of persistence of attacks or of subclinical inflammation and may reduce the dose in stable patients with no attacks for more than 5 years and no elevated acute phase reactants (APR). Biological treatments including IL-1 inhibitors are added when compliant patients fail to respond to the maximum tolerated dose of colchicine though colchicine should be continued in these patients. NSAIDs may be used in FMF patients who presented with acute attacks, exertional leg pain, and/or protracted febrile myalgia. FMF patients with AA amyloidosis warrant using the maximal tolerated dose of colchicine with or without biologics as needed to avoid the fatal progression of the amyloid deposition [13]. Hemodialysis and renal transplantation are considered for the treatment of ESRD. Other interventions are targeted towards the involved organs [14].

FMF complications include AA amyloidosis, small bowl obstruction, and infertility. Colchicine treatment is effective in preventing these complications [8]. The most devastating FMF complication and a major cause of death in patients with FMF is the progressive AA amyloidosis [1, 15]. AA amyloidosis is common in FMF patients with frequency rates ranging from 8.6% to 12.9 [16, 17].

The development risk of AA amyloidosis is higher in patients with M694V MEFV gene mutation, alpha allele of the type 1 serum amyloid A protein, male gender, eastern Mediterranean origin, and a positive family history of AA amyloidosis [18, 19]. This patient had significant risk factors to develop AA amyloidosis included male gender, M694V MEFV gene mutation, a positive family history, suboptimal daily dose of colchicine, and the patient's medication noncompliance. These factors probably increased the risk to develop the AA amyloidosis that ended up with renal failure at the age of 17 years. Due to the fact that the other sick family members are at the risk to develop amyloidosis or renal disease, they are maintained on a more frequent follow-up schedule.

AA amyloidosis can lead to different organ dysfunctions. Although amyloid proteins usually deposit in kidneys causing ESRD, in the gastrointestinal tract causing malabsorption, in the liver and spleen causing hepatosplenomegaly, in testes causing azoospermia and infertility, and in the heart causing heart failure, both adrenal and thyroid glands can be affected as well [20, 21].

AA amyloidosis maybe prevented by lifelong daily colchicine treatment. In colchicine treatment adherent patients, the incidence rate of proteinuria development was 1.7% while it was 49% in nonadherent patients [22]. Other studies found that the risk of AA amyloidosis increased to 60% and 75% in untreated Turkish and Jewish patients aged 40 and above, respectively [19, 23]. Renal AA amyloidosis usually presents with proteinuria and kidney injury and maybe complicated by ESRD. Other manifestations of AA amyloidosis in FMF patients present mainly due to the increased life expectancy in dialysis and renal transplant patients [19, 23]. Colchicine treatment can prevent the recurrent disease in transplant kidney patients [12]. Our patient had the transplanted kidney for 2 years and maintained on daily colchicine and immunosuppressants. The patient did not manifest any recurrent disease.

The presence of both adrenal crisis and clinically detectable goiter is an extremely rare complication of amyloidosis secondary to FMF [3]. The triad amyloidosis involvement of the adrenals, thyroid, and heart was not reported previously.

Although rarely adrenal glands can be affected in FMF patients and may lead to an adrenal crisis, it is significant to detect and treat this condition promptly. Its prevalence is not studied [3, 24]. Adrenal crisis patients present with unexpected hypotension, nausea, vomiting, abdominal pain, and diarrhea. Laboratory of adrenal crisis patients shows hyponatremia, hyperkalemia, and hypoglycemia. Sometimes, it may be very difficult to distinguish between the adrenal crisis and FMF attacks due to the huge heterogeneity of the clinical manifestations for the FMF [3].

Cortisol level, CBC, ABGs, ACTH stimulation test, imaging studies, and serum antibodies screening for Addison disease may all be required to establish a diagnosis of adrenal crisis. However, FMF patients may have a challenging test result interpretation due to the negative correlation between the interleukin-6 and the level of cortisol during febrile attacks. In our patient, the ACTH stimulation test result and the atypical serum electrolytes maybe best explained by the cortisol intake status of the patient, and we did not stop the cortisol intake before test performance due to the theoretical risk of the transplant rejection. Since an adrenal crisis can have a potential risk of morbidity and mortality, it should be differentiated from the far more common FMF attacks. When patients presented with different tissue-diagnosis amyloidosis with adrenal crisis, the amyloidosis-induced primary adrenal disease should be considered. Corticosteroids and mineralocorticoids are the standard treatment [20, 21].

The thyroid gland is commonly affected by amyloidosis in FMF patients. However, amyloid goiter is rarely detected clinically with a prevalence of 0.27% [1]. Amyloid goiter patients are usually euthyroid but can have hypothyroid or hyperthyroid function status. Other patients can present with the euthyroid sick syndrome and some may develop mass effects as dyspnea, dysphagia, and/or hoarseness, and maybe painful. Thyroid US shows hypoechoic nodules or diffuse multinodular goiter. Thyroid FNA is the standard diagnostic approach for amyloid goiter [25].

Cardiac amyloidosis secondary to FMF is an uncommon but a bad prognostic factor in patients with FMF. Cardiac amyloidosis can present with restrictive cardiomyopathy, valvular heart disease, coronary artery disease, and/orpericardial disease. Echocardiography and/or cardiac tissue biopsy is the standard diagnostic approach for cardiac amyloidosis [26].

## 4. Conclusion

Familial Mediterranean fever (FMF) may be complicated by AA amyloidosis that can lead to different organ dysfunctions. Adrenal involvement is rare and the same is for the thyroid gland. The coexistence involvement for both glands is an extremely rare manifestation. In the current report, we presented for the first time in the literature a triad of adrenal, thyroid, and cardiac amyloidosis secondary to FMF. Adrenal amyloidosis can lead to an adrenal crisis which can closely mimic the acute FMF inflammatory attacks.
